# An Increase in Mucin2 Expression Is Required for Colon Cancer Progression Mediated by L1

**DOI:** 10.3390/ijms241713418

**Published:** 2023-08-30

**Authors:** Arka Saha, Nancy Gavert, Thomas Brabletz, Avri Ben-Ze’ev

**Affiliations:** 1 Department of Molecular Cell Biology, Weizmann Institute of Science, Rehovot 7610001, Israel; arka.saha@weizmann.ac.il (A.S.); nancy.gavert@weizmann.ac.il (N.G.); 2 Department of Experimental Medicine I, Nikolaus-Feibiger-Center for Molecular Medicine, University of Erlangen-Nuernberg, 91054 Erlangen, Germany; thomas.brabletz@fau.de

**Keywords:** Mucin2, L1, colon cancer, tumorigenesis, metastasis

## Abstract

An induction in the expression of the cell adhesion receptor L1, a Wnt target gene, is a characteristic feature of Wnt/β-catenin activation in colon cancer cells at later stages of the disease. We investigated the proteins secreted following L1 expression in colon cancer cells and identified Mucin2 among the most abundant secreted proteins. We found that suppressing Mucin2 expression in L1-expressing colon cancer cells inhibits cell proliferation, motility, tumorigenesis, and liver metastasis. We detected several signaling pathways involved in Mucin2 induction in L1-expressing cells. In human colon cancer tissue, Mucin2 expression was significantly reduced or lost in the adenocarcinoma tissue, while in the mucinous subtype of colon cancer tissue, Mucin2 expression was increased. An increased signature of *L1*/*Mucin2* expression reduced the survival rate of human colon cancer patients. Thus, induction of Mucin2 expression by L1 is required during mucinous colon cancer progression and can serve as a marker for diagnosis and a target for therapy**.**

## 1. Introduction

Overactivation of the Wnt/β-catenin signaling pathway and its downstream target genes by the β-catenin-T-cell factor complex is a hallmark of human colon cancer development [1,2,3]. We previously detected members of the L1 family of transmembrane cell adhesion receptors (L1 and NrCAM) as target genes of β-catenin-T-cell factor transactivation in colon cancer cells [4,5]. L1 is mostly known for its key roles in human brain development through its involvement in the dynamic processes of axonal elongation, fasciculation, and pathfinding [6,7,8]. Point mutations all along the L1 molecule that inactivate it can result in severe developmental brain diseases such as mental retardation, aphasia, shuffling gait, adducted thumbs (MASA syndrome), X chromosome-linked hydrocephalus, and L1 syndrome [7,9,10,11,12,13,14]. In human colon cancer tissue, L1 is exclusively expressed at the invasive edge of the tumor [5]. The analysis of genes and proteins whose secretion is induced by L1 expression in colon cancer cells identified Mucin2 among the most abundant proteins secreted by L1-overexpressing cells [15]. Mucin2 belongs to the heavily glycosylated high molecular weight family of glycoproteins comprising more than 20 members [16,17,18]. The intestinal mucus layer consists mainly of Mucin2, a major mucin synthesized and secreted by goblet cells [19,20,21]. It comprises most of the secreted mucinous proteins around the colon cancer tissue [22,23,24] and plays a crucial role in forming the mucinous layer around the normal colonic epithelium that protects it from pathogens and mechanical insults [25,26,27,28]. The altered expression of Mucin2 is often a characteristic feature of colon cancer progression [16,29,30]. In this study, we investigated the role of Mucin2 in L1-mediated colon cancer development. 

## 2. Results

### 2.1. Induction of Mucin2 Expression in Human Colon Cancer Cells and the Secretome of L1-Overexpressing Cells 

**Figure 1:**
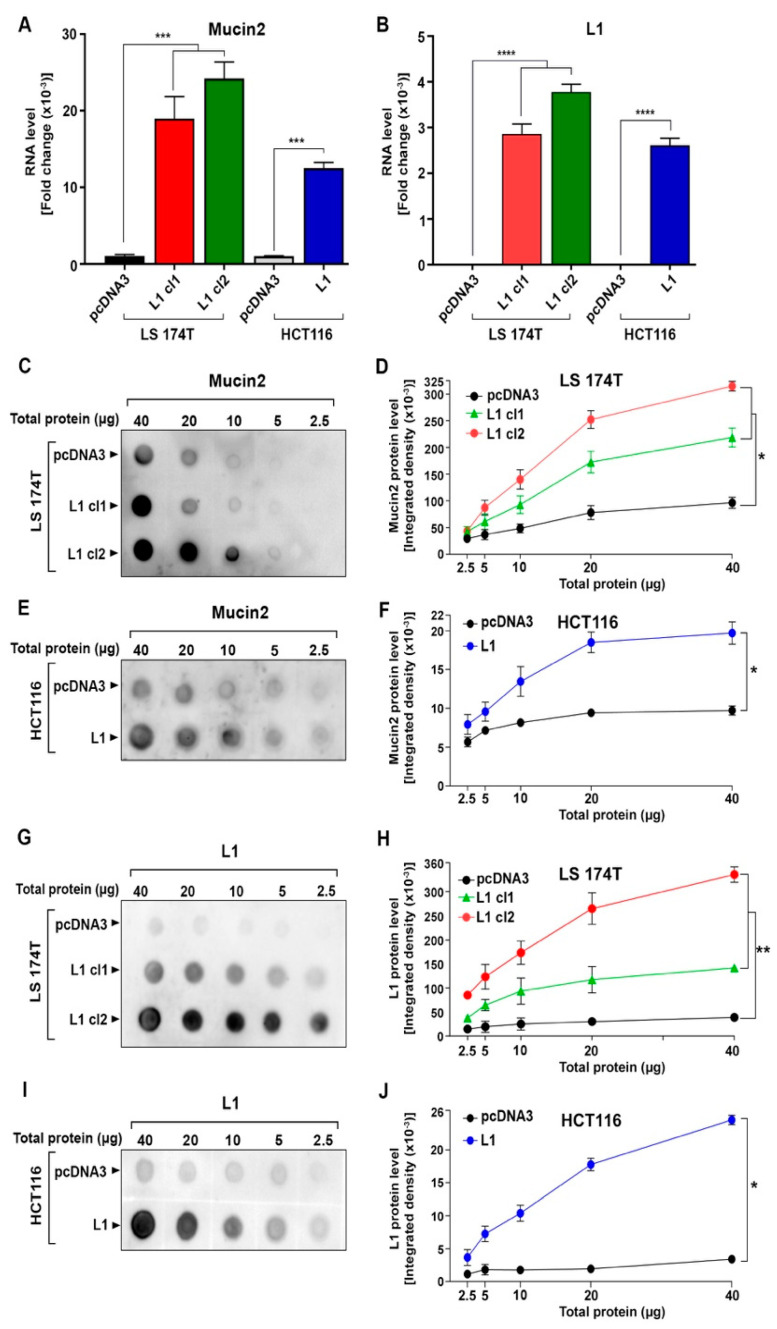
Induction of Mucin2 expression in human colon cancer cell lines overexpressing L1. The level of *Mucin2* (**A**) and *L1* (**B**) RNA was determined in LS 174T (cl1 and cl2) and HCT116 colon cancer cells transfected with either *L1* or pcDNA3 (control) by RT-PCR. (**C**) Dot blot analysis of Mucin2 in LS 174T and (**E**) in HCT116 cells and L1 protein (**G**,**I**) using serial dilutions of whole cell extracts from these colon cancer cell lines with the respective antibodies. The graphs in (**D**,**F**,**H**,**J**) show Mucin2 and L1 levels at each protein concentration loaded from triplicate experiments. The significance in the changes of dot intensities were calculated by the student’s unpaired *t*-test. (* *p* < 0.05, ** *p* < 0.01, *** *p* < 0.001, **** *p <* 0.0001).

Our previous proteomic study interrogating the secreted proteins from L1-overexpressing colon cancer cells identified Mucin2 among the most highly expressed proteins after L1 transfection [15]. We wished to determine whether *Mucin2* RNA and protein were increased by L1 in different colon cancer cell lines and used LS 174T and HCT116 human cells to assess *Mucin2* expression in these cells after L1 overexpression. The results demonstrate that in both LS 174T and HCT116 colon cancer cells overexpressing *L1* RNA (Figure 1B), there is a dramatic increase in the *Mucin2* RNA level (Figure 1A). Quantitative dot blot analysis of Mucin2 protein levels with anti-Mucin2 antibodies detected a rise in Mucin2 protein (Figure 1C,E) in both colon cancer cell lines overexpressing L1 (Figure 1D,F). In LS 174T clones expressing a higher level of L1 (Figure 1H), there was also a greater increase in Mucin2 level (Figure 1D). 

**Figure 2:**
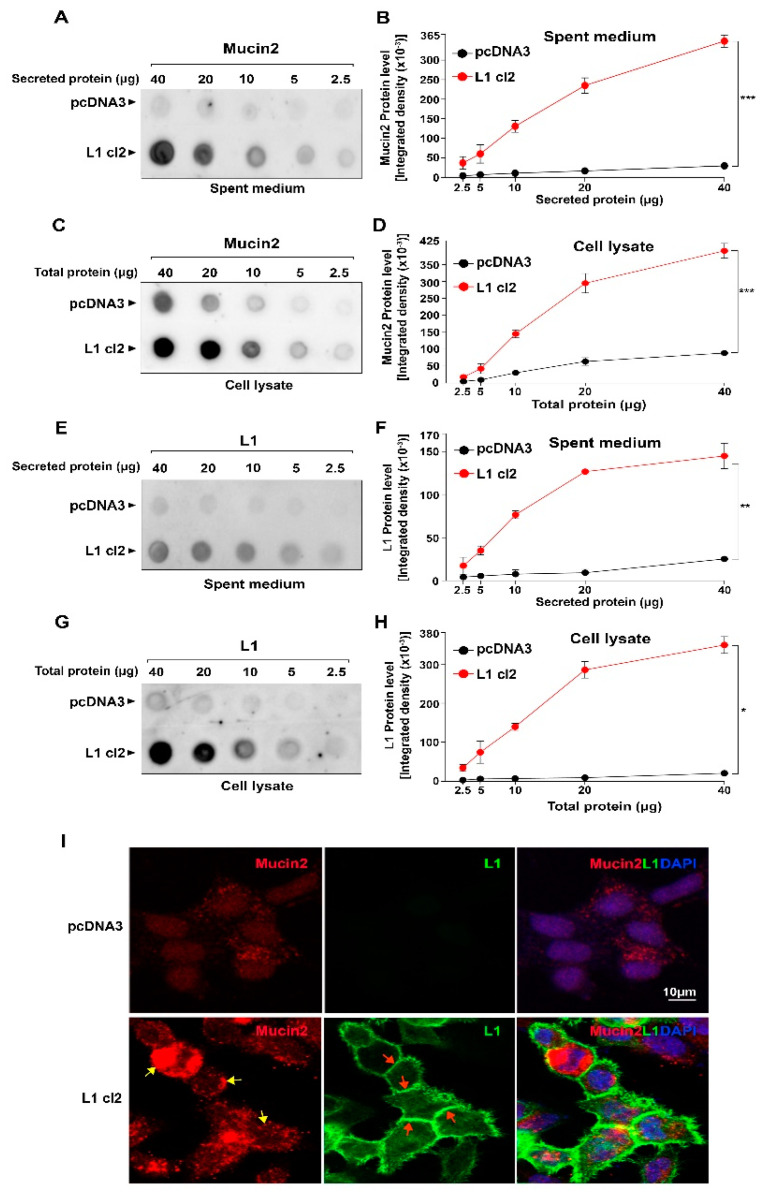
Increased Mucin2 secretion into the culture medium by cells overexpressing L1. The levels of Mucin2 (**A**,**B**) in the culture medium and in cell lysates (**C**,**D**) were determined in LS 174T cells transfected with L1, or with the control pcDNA3 plasmid, by quantitative dot blot analysis of serial dilutions of both cell lysates and the culture medium (spent medium). The levels of L1 in the medium (**E**,**F**) and in cell lysates (**G**,**H**) of the same cells. (**I**) Immunofluorescence localization of Mucin2 (red staining, yellow arrows showing secretory granules) and L1 (green staining, red arrows marking cell-cell contact sites) in LS 174T cells transfected with either the pcDNA3 plasmid or an L1-expressing construct. Nuclei were stained with DAPI (blue). (* *p* < 0.05, ** *p* < 0.01, *** *p* < 0.001).

A higher level of Mucin2 was seen both in the cells (Figure 2C,D) and secreted into the medium (Figure 2A,B) of colon cancer cells overexpressing L1 compared to control pcDNA3-transfected cells. This was also seen for L1 expression (Figure 2E–H). Immunofluorescent staining of Mucin2 in colon cancer cells revealed that in L1-overexpressing cells, there is intense staining of Mucin2 in secretory granules in the cytoplasm (Figure 2I, red staining, yellow arrows), while L1 was detected at cell-cell contact sites (Figure 2I, green staining, red arrows). In contrast, the control pcDNA3-transfected colon cancer cells displayed only weak staining of Mucin2 and no significant staining for L1 (Figure 2I). These results suggest that L1 expression in human colon cancer cells confers increased RNA and Mucin2 protein levels in these cells and elevated secretion of Mucin2 into the culture medium of L1-expressing cells.

### 2.2. Isolation of L1-Expressing Colon Cancer Cell Clones with Suppressed Mucin2 Levels 

**Figure 3:**
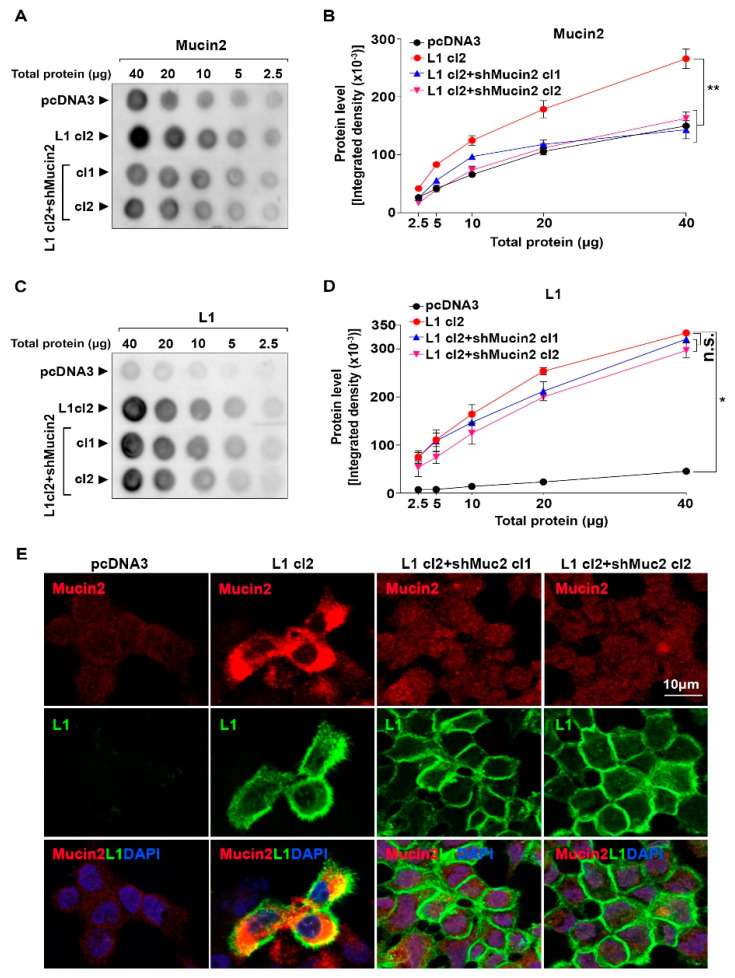
Isolation of L1-expressing colon cancer cell clones in which Mucin2 levels were suppressed. (**A**,**B**) L1-overexpressing LS 174T clones in which the expression of Mucin2 was reduced by shRNA to Mucin2 were isolated (L1 + shMucin2 cl1 and cl2), and the level of Mucin2 determined by quantitative dot blot analysis and compared to Mucin2 levels in L1- and pcDNA3-transfected cells. (**C**,**D**) The expression of L1 was determined in the LS 174T cell clones described in (**A**). (**E**) Mucin2 (red) and L1 (green) localization was determined by immunofluorescent staining with the respective antibodies to Mucin2 and L1. Nuclei were stained with DAPI (blue). (* *p* < 0.05, ** *p* < 0.01, n.s. is statistically non-significant).

To analyze the possible role of Mucin2 in L1-mediated colon cancer cell tumorigenesis, we isolated L1-expressing colon cancer cell clones in which the level of Mucin2 was reduced by shRNA constructs against *Mucin2*. In these colon cancer cell clones with reduced Mucin2 levels (Figure 3A,B, shMucin2 cl1, and cl2), the expression of L1 was not significantly affected (Figure 3C,D, n.s.). The immunofluorescence analysis of these colon cancer cell clones revealed a dramatic reduction in Mucin2 staining (Figure 3E, L1 + shMucin2 cl1, and cl2), but without an apparent change in L1 staining that was localized at cell–cell contact sites (Figure 3E, compare L1 to L1 + shMucin2 cl1 and cl2).

### 2.3. Suppression of Mucin2 Levels Inhibits the Motile, Growth, Tumorigenic, and Metastatic Properties of L1-Expressing Colon Cancer Cells

**Figure 4:**
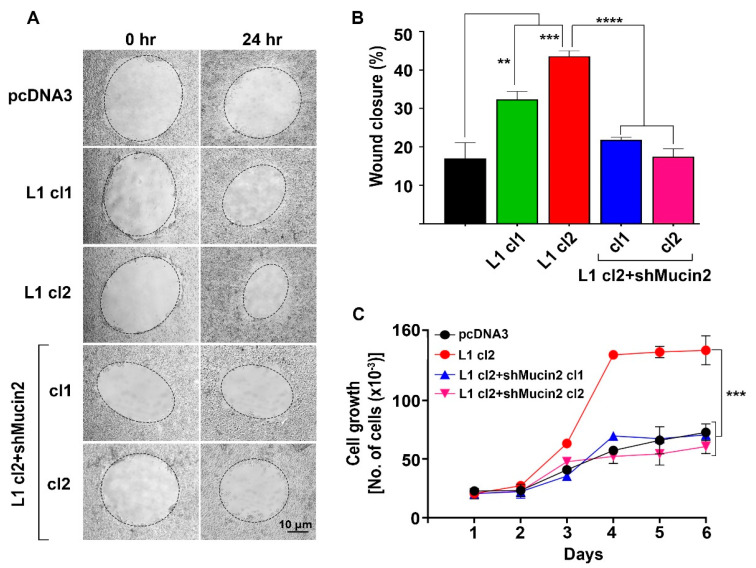
Reduced cell motility and proliferation in L1-expressing colon cancer cells with suppressed Mucin2 levels. (**A**,**B**) Decreased motility determined by the closure of “scratch” wounds 24 h after wounding the monolayers of LS 174T cell clones expressing L1 + shMucin2 compared to L1 cl2 and pcDNA3 transfected cells. (**C**) Cell proliferation was measured during six days in the various cell clones described in (**B**) by seeding the same cell number (in triplicates), culturing the cells in the presence of 0.5% serum, and determining their number each day. (** *p* < 0.01, *** *p* < 0.001, **** *p <* 0.0001).

We have used the L1-expressing colon cancer cell clones described in Figure 3 with reduced Mucin2 levels and determined their motile properties by the rate of closure of “scratch” wounds introduced in a monolayer. The results shown in Figure 4A,B demonstrate significantly lower motility in clones of L1-expressing cells with reduced Mucin2 levels. Next, we determined the proliferation of these cell clones under stress (in the presence of 0.5% serum) and found a reduced proliferation rate in L1 + shMucin2 colon cancer cell clones (Figure 4C, L1 cl2 + shMucin2 cl1 and cl2).

**Figure 5:**
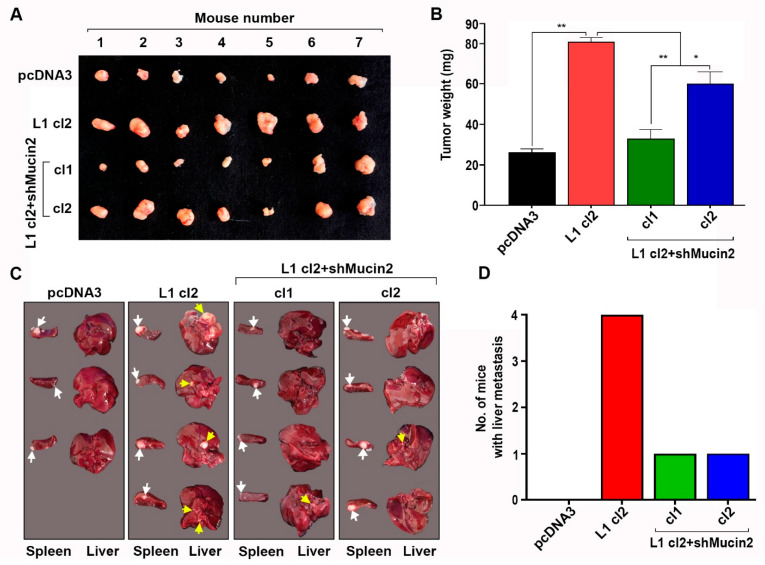
Tumor growth and liver metastasis of colon cancer cells are suppressed when Mucin2 levels are reduced in L1-expressing cells. (**A**) Groups of seven mice (five-week-old) were injected subcutaneously with the LS 174T cell clones described in Figure 3, and the tumor growth (weight) was determined after two weeks (**B**). (**C**,**D**) Groups of four mice were injected in the tip of their spleen with the cell lines described in (**A**), and tumor growth in the spleen (white arrows) and the extent of liver metastasis (yellow arrows) was determined after five weeks (photographed livers and spleens). (* *p <* 0.05, ** *p* < 0.01).

Next, we analyzed the effect of changes in Mucin2 levels on the tumorigenic properties of these cell clones “in vivo.” Mice were injected subcutaneously with cells of the different clones, tumors were excised, and their weight was determined after three weeks. As shown in Figure 5A,B, the suppression of Mucin2 in L1-expressing cells resulted in decreased tumor growth. The expression of Mucin2 remained low in the tumor tissue of L1 + shMucin2-injected cells after 3 weeks of growth in mice (Supplementary Figure S1). 

The metastatic ability of L1-expressing colon cancer cells in which Mucin2 levels were suppressed was also determined by injecting the cells in the spleen of nude mice and following the formation of metastases in the liver after six weeks. The results showed a dramatic reduction in the metastatic potential of L1-transfected cells in which Mucin2 levels were reduced (Figure 5C,D, L1 cl2 + shMucin2 cl1, and cl2). Together, these results suggest that decreased Mucin2 expression in L1-transfected colon cancer cells inhibits the motility, proliferation, tumorigenesis, and liver metastasis of these cells.

### 2.4. Signaling Pathways Involved in Mucin2 Induction by L1 in Colon Cancer Cells

**Figure 6:**
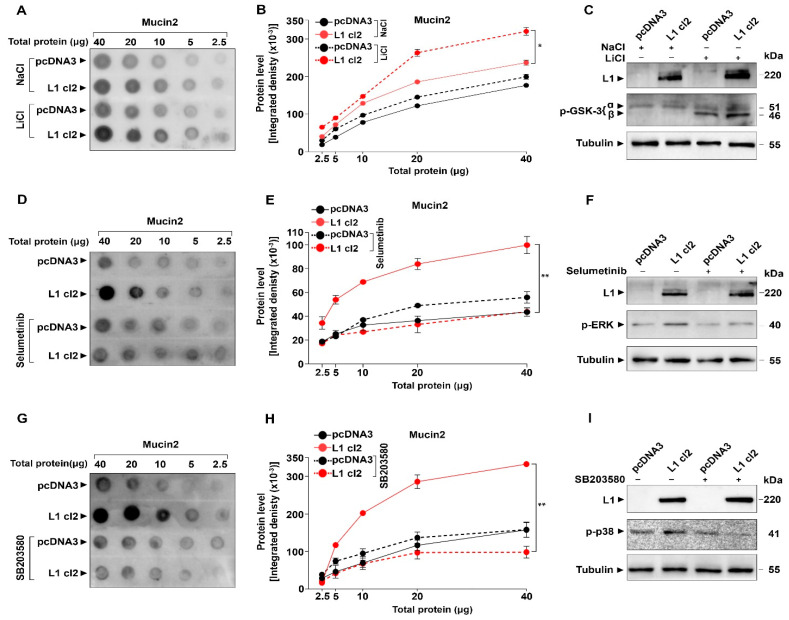
Wnt, ERK, and p38 signaling are involved in the induction of Mucin2 by L1. Wnt/β-catenin signaling was induced by inhibiting GSK3β ((**C**), a key component of the β-catenin destruction complex) using LiCl (NaCl served as negative control) and the level of Mucin2 was determined by dot blotting using the anti Mucin2 antibodies (**A**,**B**) and that of phosphorylated GSK3β by western blotting with p-GSK3 antibody. ERK inhibition was achieved by decreasing its phosphorylation using Selumetinib (**F**). The level of Mucin2 was determined in the treated and untreated cells by quantitative dot blotting (**D**,**E**). The inhibition of signaling by the p38 pathway was achieved by inhibiting its phosphorylation with SB203580 (**I**). The levels of Mucin2 in the treated and untreated cell clones was determined by quantitative dot blotting (**G**,**H**). Tubulin served as a loading control. (* *p* < 0.05, ** *p* < 0.01).

**Figure 7:**
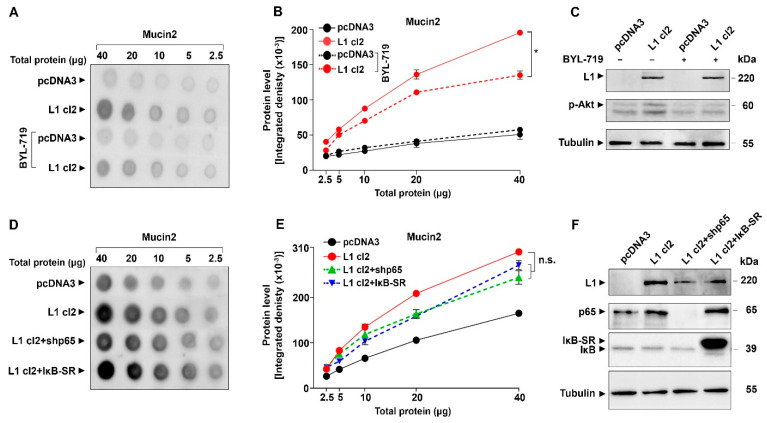
Akt but not NF-κB signaling is involved in Mucin2 induction by L1. The level of Mucin2 was determined by quantitative dot blotting (**A**,**B**) in cells with or without inhibition of Akt with BYL-719. The level of Akt phosphorylation was determined using an anti-p-Akt antibody (**C**). The expression of Mucin2 was determined in LS 174T cells expressing L1 in which NF-κB signaling was blocked using the NF-κB inhibitor IκB-SR (L1 cl2 + NFκB-SR) and in cells expressing shRNA to p65 (L1 cl2 + shp65) (**D**,**E**). The levels of L1, p65, IκB-SR, and IκB were determined by western blotting (**F**). Tubulin served as a loading control. (* *p* < 0.05, n.s., not significant statistically).

We wished to determine which signaling pathways are regulating the induction of Mucin2 in L1-expressing colon cancer cells. In previous studies, we have shown that the Wnt/β-catenin pathway is involved in the regulation of other genes by L1 [15]. We stimulated this pathway by inhibiting the β-catenin destruction complex with lithium chloride (LiCl) that induces increased GSK3β phosphorylation/inactivation (Figure 6C). We found an increase in Mucin2 level in L1-expressing cells treated with LiCl (Figure 6A,B), indicating the involvement of Wnt/β-catenin signaling in L1-mediated Mucin2 expression. Next, we inhibited the ERK pathway with Selumetinib [31], which decreases the level of p-ERK (Figure 6F), and found an inhibition in Mucin2 expression in L1-expressing colon cancer cells (Figure 6D,E), suggesting an involvement of ERK signaling in Mucin2 expression by L1. We also found that reducing p38 activity by the p38 inhibitor SB20358 [32] reduces the Mucin2 expression in L1-expressing colon cancer cells (Figure 6G,H), which indicates the involvement of the p38-MAPK pathway in the induction of Mucin2 by L1. We have also examined the involvement of the Akt pathway in the induction of Mucin2 by L1 using the BYL-719 inhibitor of Akt activation/phosphorylation [33] (Figure 7C) and found that a reduced Akt activity results in the inhibition of Mucin2 expression in L1-expressing colon cancer cells (Figure 7A,B). Since our previous studies indicated that a number of genes induced by L1 overexpression in colon cancer cells involve the NF-κB pathway [34,35], we have also examined the expression of Mucin2 under conditions when this pathway was inhibited by the expression of shRNA against the p65 subunit of NF-κB (shp65) and in cells where the pathway was blocked using the IκB-SR (Figure 7F). The results shown in Figure 7D,E demonstrate that Mucin2 expression in L1-expressing cells was not significantly affected when NF-κB signaling was blocked. The small decrease in Mucin2 (statistically not significant) in the L1 + shp65 and L1 + IκB-SR cell clones resulted from the lower level of L1 in these cells as compared to L1 cl2 (Figure 7F). Taken together, these results indicate that numerous pathways are involved in the regulation of Mucin2 induction by L1. These pathways apparently do not include NF-κB signaling. 

### 2.5. Localization of Mucin2 in Normal Mucosa and the Adjacent Colon Cancer Tissue and a Correlation of High Mucin2/L1 Signature with Deceased Patient Survival

**Figure 8:**
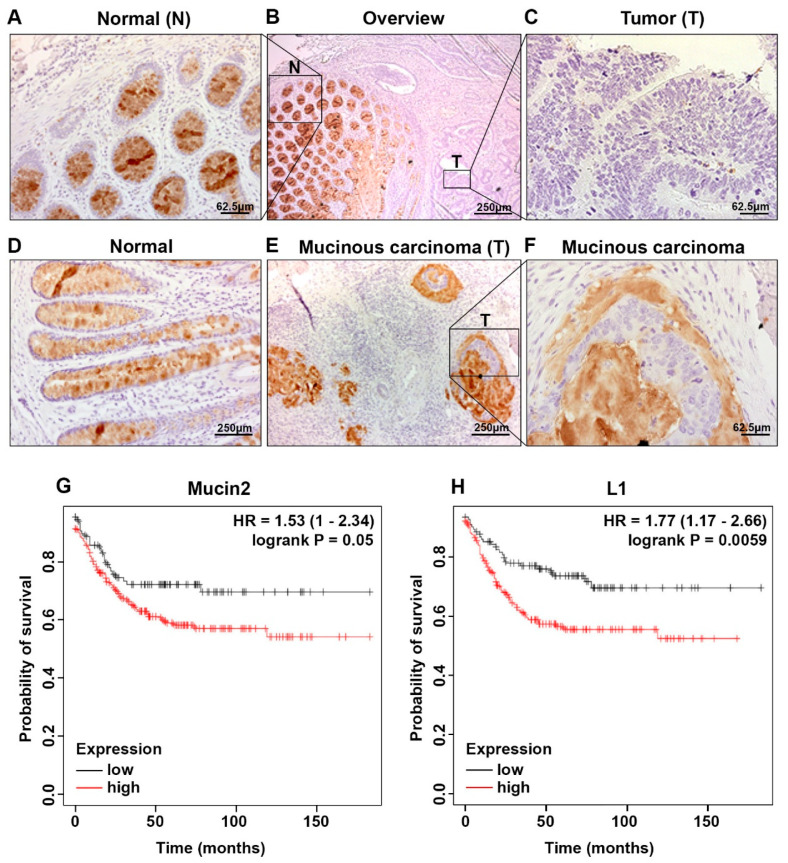
Localization of Mucin2 in normal human mucosa and colon cancer tissue and the correlation between L1 and Mucin2 expression and colon cancer patient survival. Thirty-eight formaldehyde-fixed and paraffin-embedded human colon cancer tissue samples were processed by immunohistochemistry with antibodies to Mucin2. (**A,D**) Normal colon mucosa expressed Mucin2 in goblet cells [Normal (N)]. (**B**,**C**) The adjacent CRC tissue (T) displayed a complete loss, or much reduced, staining for Mucin2 in 63% of the cases. (**E**,**F**) The remaining 38% of the CRC tissue samples displayed a mucinous phenotype (T). (**G**,**H**) Kaplan-Meier analysis of the survival probability in high (red) and low (black) *Mucin2* (**G**) and *L1* (**H**) expressing colon cancer patients.

We wished to determine the localization of Mucin2 in human colon cancer tissue and the adjacent normal mucosa. Sections from 38 paraffin-embedded human colon cancer tissue samples were analyzed by immunohistochemistry with an antibody to Mucin2. In the normal mucosa, Mucin2 was expressed in goblet cells (Figure 8A,B,D). The staining for Mucin2 was negative or very weak in 63% of the colon cancer tissue samples (Figure 8C). In comparison, 38% of the cases were positive for Mucin2, with the tumor tissue displaying a mucinous phenotype (Figure 8E,F). Finally, we determined the survival probability of colon cancer patients expressing a high level of either Mucin2 and L1 and found that a high *Mucin2*/*L1* signature results in decreased survival of colon cancer patients. The results demonstrated a dramatic change in Mucin2 expression and phenotype in colon cancer tissue and indicated that a high expression of Mucin2 and L1 results in decreased survival probability.

## 3. Discussion

This study shows that increased *Mucin2* expression and secretion in human colon cancer cells is required for L1-mediated colon cancer progression. Suppression of Mucin2 levels in L1-expressing colon cancer cells inhibited cell proliferation, motility, tumor growth, and liver metastasis in mice. Mucin2 thus joins a long list of secreted proteins (mainly pericellular and ECM-associated) whose increased expression in L1-overexpressing colon cancer cells is required for colon cancer progression [15,34,35]. 

Our immunohistochemistry results agree with previous studies suggesting that increased expression of Mucin2 is considered a marker for mucinous colon cancer, a subtype of colon cancer [22,36,37,38]. Our results documenting a decrease or complete loss of Mucin2 in non-mucinous adenocarcinoma of the colon also agree with studies showing that low levels of Mucin2 correlate with colon cancer progression [16,39,40,41]. The significance of increased L1 and Mucin2 expression in colon cancer cells is relevant to cancer patient survival, as suggested by our demonstration that a decreased survival probability is apparent in patients with high levels of *Mucin2* and *L1* expression.

The cell adhesion receptor L1 was shown to signal in a variety of cancer cell types and in neuronal cells by different signaling pathways [42,43,44,45,46], and Mucin2 expression is regulated by several signaling pathways, including Ras, ERK, PKC, PI3K, and NF-κB signaling [47,48]**.** In this study, we showed that the mechanisms whereby L1 induces Mucin2 expression involve various pathways, including Wnt/β-catenin, ERK, p38, and Akt. Since we have shown that L1 induces several L1-target genes via the NF-κB pathway [34,35,49], we asked whether NF-κB is involved in Mucin2 induction by L1. Our results do not support a significant involvement of NF-κB in the L1-mediated induction of Mucin2, since the higher levels of Mucin2 in L1-expressing colon cancer cells remained high also after NF-κB signaling was blocked in these cells. Given the additional pathways already identified in L1-mediated signaling in colon cancer cells [15,50], further studies are required to determine the relative contributions of each pathway to Mucin2 induction by L1 expression.

## 4. Materials and Methods

### 4.1. Cell Culture and Reagents

Colon cancer cell lines LS 174T (ATCC CL-188) and HCT116 (ATCC CCL-247) were grown in RPMI-1640 medium (Gibco, Thermo Fisher Scientific, Paisley, UK) containing 10% FBS (Gibco) and 1% penicillin/streptomycin solution (Biological Industries, Beit-Haemek, Israel). LS 174T-L1, HCT116-L1 cells, L1 + shp65, and L1 + IκB-SR cells were cultured in RPMI-1640 medium supplemented with G418 (800 µg/mL) (MilliporeSigma, Burlington, MA, USA). LS 174T-L1 + shMucin2 clones were maintained in selective RPMI 1640 medium containing G418 (800 µg/mL) (Sigma-Aldrich, St. Louis, MO, USA) and puromycin dihydrochloride (5 µg/mL) (MilliporeSigma). 

LS 174T pcDNA3 (empty vector) and LS 174T-L1 cells were treated for 24 h with 5 µM of the MEK inhibitor Selumetinib (Selleck Chemicals, Sylvanfield Drive, Houston, TX, USA) [51,52] and with 5 µM of the PI3K inhibitor BYL-719 (MedChemExpress, Monmouth Junction, NJ, USA) [52]. LS 174T pcDNA3 (empty vector) and LS 174T-L1 cells were also treated with 10 µM of the p38-MAPK inhibitor SB203580 (S1076, Selleck Chemicals) for 24 h [53]. SB203580 was obtained from Prof. Rony Seger (Department of Immunology and Regenerative Biology, Weizmann Institute of Science, Rehovot, Israel). A 30 mM LiCl (MilliporeSigma) was used as glycogen synthase kinase 3 beta (GSK3β) inhibitor [15]. Cells treated with 30 mM sodium chloride (NaCl) (MilliporeSigma) served as a control [15].

### 4.2. Plasmids

ShRNA to *Mucin2* sequences were prepared using pSUPER.puro according to the manufacturer’s instruction (pSUPER.puro RNAi System, OligoEngine, WA, USA). The shRNA to *Mucin2* target sequences are presented in Table 1.

### 4.3. Transfection, Cell Proliferation, and Motility Assays

LS 174T-*L1* cells were transfected with shRNA to *Mucin2* using the Xfect™ transfection reagent (TaKaRa Bio USA Inc., San Jose, CA, USA) according to the manufacturer’s instructions [54]. For cell proliferation assay, 5000 cells were cultured in 12-well plates in triplicates containing RPMI-1640 medium containing 0.5% FBS. Cell proliferation was determined over a period of six days by the cell counting method using a hemocytometer as described [54], and the rate of proliferation was plotted using the GraphPad Prism 9 software (San Diego, CA, USA, www.graphpad.com, accessed on 1 March 2023).

Cell motility was determined by the “scratch wound” assay, as previously described [54]. Briefly, 10^5^ cells were seeded in 12-well plates and cultured to obtain a confluent monolayer. Cell proliferation was inhibited using a medium supplemented with 2.5 μg/mL Mitomycin C. Micropipette tips were used to introduce the “wounds” in the monolayer. Images of the wound were taken at 0 h and 24 h, and the change in the surface area of the wound was determined using the FIJI software (v.1.53c, NIH, Bethesda, MD, USA). The percentage of wound closure was determined using the FIJI software graphs, which were plotted using the GraphPad Prism 9 software. 

### 4.4. Immunofluorescence

For immunofluorescence assays, poly-L-lysine-coated coverslips were used to grow the cells. Cells were permeabilized with 0.5% Triton X-100 and 4% paraformaldehyde was used as a fixative. Blocking was carried out with 5% horse serum in PBS and the cells were incubated with mouse anti-L1 (Santa Cruz Biotechnology Inc., Dallas, TX, USA) diluted 1:200, and rabbit anti-Mucin2 antibodies diluted 1:200 (provided by Prof. D. Fass, Department of Chemical and Structural Biology, Weizmann Institute of Science, Israel 7610001) [55]. Cy3-tagged goat anti-rabbit IgG (Jackson Immunoresearch Laboratories, West Grove, PA, USA) and Alexa Flour-688 tagged goat anti-mouse IgG (ABCAM, Trumpington, Cambridge, UK) secondary antibodies were used at a dilution of 1:5000. Next, 5 µg/mL of 4′-6-diamidino-2-phenylindole (DAPI, Sigma-Aldrich, St. Louis, MO, USA) was used to stain nuclei. The Zeiss LSM 980 confocal microscope was used to visualize signals and images were captured using the ZEN imaging software v3.7 (Carl Zeiss Microscopy GmbH, Jena, Germany) and analyzed using the FIJI software.

### 4.5. Western and Dot Blotting

Dot blotting was performed to detect total Mucin2 and L1 protein levels in cells. The Radioimmunoprecipitation assay (RIPA) lysis buffer, supplemented with 1% protease inhibitor cocktail, was used to prepare cell lysates. Total protein concentration was determined by the Pierce biocinchonic acid (BCA) protein assay (Thermo Fisher Scientific, Paisley, UK) according to the manufacturer’s instructions [34]. For dot blots, cell lysates were serially diluted between 40 μg to 2.5 µg of protein and blotted on a 0.45 µm porous nitrocellulose membrane (Bio-Rad Laboratories, Haifa, Israel). 

Western blotting was performed on whole cell lysates of pcDNA3 and L1-expressing cells to determine the effects of the kinase inhibitors Selumetinib, SB203580, BYL-719, and LiCl. Briefly, 40 μg protein was resolved by 12% SDS-PAGE and transferred to 0.45 µm nitrocellulose membranes (Bio-Rad Laboratories).

The membranes were dried and blocked with 5% bovine serum albumin (BSA) in phosphate buffered saline (PBS) containing 0.5% Tween-20 (Bio-Basic Canada, Inc., Markham, ON, Canada) and probed with the following antibodies at room temperature for 2 h: rabbit anti-L1 antibody (provided by Prof. Vance Lemmon, University of Miami, Miami, FL, USA) diluted 1:2000 and rabbit anti-Mucin2 antibody (provided by Prof. Deborah Fass, Department of Chemical and Structural Biology, Weizmann Institute of Science, Israel) diluted 1:3000; rabbit anti-phospho-GSK3α/β (Ser21/9) (Cell Signaling Technologies, Beverly, MA, USA) diluted 1:2000; anti-phospho-p38 (Sigma-Aldrich, St. Louis, MO, USA), obtained from Prof. Yosef Yarden, Department of Immunology and Regenerative Biology, Weizmann Institute of Science, Rehovot, Israel, diluted 1:10,000; mouse anti-phospho ERK1/2 (Sigma-Aldrich), obtained from Prof. Rony Seger, Department of Immunology and Regenerative Biology, Weizmann Institute of Science, Rehovot, Israel, diluted 1:10,000; rabbit anti-phospho-Akt, s473, 4060, clone D9E (Cell Signaling Technologies, Beverly, MA, USA, from Dr. Nancy Gavert) diluted 1:2000, anti-rabbit NFκB p65, sc-109 (Santa Cruz Biotechnology, Inc., Dallas, TX, USA) diluted 1:2000; and anti-rabbit phospho-IκBα #2859 (Cell Signaling Technologies Inc., Danvers, MA, USA) and anti-mouse anti-β-tubulin (Sigma-Aldrich), diluted 1:5000. The membranes were incubated at room temperature for 2 h with goat anti-rabbit and anti-mouse HRP-conjugated secondary antibodies (ABCAM) at a dilution of 1:2000. Membrane immunostaining was detected by the ECL method (Amersham), and the blots were imaged by the ChemiDoc MP imaging system (Bio-Rad Laboratories). Signal intensities of dots at individual protein concentrations from the dot blots were determined using the FIJI software. The integrated densities were plotted at each protein concentration of the samples used from triplicate experiments using the GraphPad Prism 9 software.

### 4.6. Quantitative RT-PCR

Isolation of total RNA from cells was performed using Bio-Tri reagent (Bio-Lab, Jerusalem, Israel) following the manufacturer’s instructions. First-strand cDNA synthesis from the isolated RNA was used to synthesize first-strand cDNA using the SuperScript™ II Reverse Transcriptase kit (Thermo Fisher Scientific, Waltham, MA, USA) by the manufacturer’s protocol [34]. Next, 200 ng/µL of cDNA were used for quantitative real-time PCR analysis with Fast SYBR™ green master mix (Applied Biosystems™, Thermo Fisher Scientific Inc., Vilnius, Lithuania) as described [54]. The primers used are described in Table 2. Gene expression was determined using the QuantStudio Design and Analysis software v1.5.1 (Thermo Fisher Scientific), and fold changes were calculated using the ΔΔCT method and plotted using the GraphPad Prism 9 software.

### 4.7. Tumor Growth and Metastasis Assays

Subcutaneous tumor growth was performed using athymic, 5-week-old male nude mice (Foxn-1nu) as previously described [34]. Briefly, 3 × 10^6^ cells suspended in 100 µL PBS were injected subcutaneously at different sites on the flanks of mice [34]. Tumor growth in mice was monitored for three weeks after injection. The experimental end point was defined as the time when tumor size reached approximately 1 cm in diameter. The tumors were excised, weighed, and photographed. A graph showing tumor weight in milligrams for each injected cell type was plotted using the GraphPad Prism 9 software. 

The metastasis assay was performed by determining the ability of cells to migrate from the spleen to the liver. Briefly, 3 × 10^6^ cells suspended in 20 µL of PBS were injected into the distal tip of the spleen of 4-week-old, athymic, male nude mice, as described [34]. Mice were anesthetized by peritoneal injection of Ketamine and Xylazine before the injection of cells into the spleen. The mice were sacrificed five weeks after injection, tumor formation at the site of injection and the presence of liver metastases in the liver were photographed. 

### 4.8. Ethics Approval

The Weizmann Institute Animal Care and Use (IACUC) ethics committee reviewed, approved, and supervised the animal studies. 

### 4.9. Immunohistochemistry and Survival Analysis

Colorectal cancer tissue samples (G2 or G3 adenocarcinomas) were recovered from the archives of the Dept. of Pathology, University of Erlangen, Germany. The retrieved samples were from patients operated on in 2003 or earlier. Patient identity was anonymized and an informed consent for usage of archive formaldehyde-fixed, paraffin-embedded material was not required at that time. The local ethics committee approved the usage of these samples for immunohistochemical analysis (approval 374-14).

Immunohistochemistry was performed on 38 paraffin-embedded human colorectal adenocarcinoma samples and adjacent normal tissues. Next, 4 mm tissue sections were deparaffinized, dehydrated, and pretreated with 10 mM citrate buffer pH 6 (citric acid monohydrate, Sigma-Merck, Darmstadt, Germany, #C-7129 and 0.05% Tween-20) at 121 °C in a pressure cooker for 22 min. Mouse monoclonal anti-Mucin2 primary antibody (1:150, #AMAB91542 Sigma-Merck, Darmstadt, Germany) diluted 1:20,000 in Dako diluent with background reducing components (#S3022 Dako, Brüsseler Str. 22, Jena, Germany) was added to the sections and incubated overnight at 4 °C. Slides were washed twice with TBS containing 0.05% Tween-20 and were developed with the EnVision System (Dako) and DAB (Cell Signaling DAB substrate kit #8059S) for visualization, according to the manufacturer’s instructions [5].

To determine the involvement of *Mucin2* and *L1* gene expression in recurrence-free survival of colon cancer cases, an online bioinformatics tool called the Kaplan–Meier plotter (https://kmplot.com/analysis/, accessed on 7 June 2023) was utilized. The Kaplan–Meier plotter online tool was used to generate survival plots for both *Mucin2* (Affymetrix ID: 204673_at) and *L1* (Affymetrix ID: 204585_s_at) expression in 297 colon cancer patients displaying distal tumor localization and stable or low MSI from the GEO, EGA and TCGA databases as described [56,57]. 

### 4.10. Statistical Analysis

Statistical analysis of the data was performed by the GraphPad Prism 9 software. The student’s non-paired *t*-test tool was used to determine the statistical significance. Results with a *p* < 0.05 were considered statistically significant and presented on the graphs with asterisks.

## 5. Conclusions

In this study, we addressed the role of Mucin2 in L1-mediated colon cancer progression. We observed high Mucin2 levels in the cell layer and spent medium of L1-expressing colon cancer cells and found that inhibiting Mucin2 expression in L1-expressing colon cancer cells inhibits their motility, proliferation, tumorigenesis, and liver metastatic capacity. The signaling pathways by which L1 induces Mucin2 involve the Wnt/β-catenin, ERK, p38, and Akt pathways. In addition, we detected a loss of Mucin2 in non-mucinous adenocarcinoma, while strong staining for Mucin2 was observed in mucinous colon cancer, indicative of the role of Mucin2 as a marker for mucinous colon cancer. A high expression of L1 and Mucin2 reduced the probability of colon cancer patient survival, suggesting an important role in colon cancer development.

In conclusion, the studies described here demonstrate that the increase in Mucin2 expression and secretion by L1 is a crucial step in colon cancer progression (especially in the mucinous subtype) and can serve in early diagnosis and in designing new targets for therapy. 

## Figures and Tables

**Table 1:** Sequences of shRNA targeted against *Mucin2* RNA.

Name	Sequence
shMucin2_1	GATCCCCGCTCTCCAATAACCACCACTTCAAGAGAGTGGTGGTTATTGGAGAGCTTTTTA
shMucin2_2	GATCCCCCGACTACAAGATACGTGTCTTCAAGAGAGACACGTATCTTGTAGTCGTTTTTA
shMucin2_3	GATCCCCACTACAAGATACGTGTCAATTCAAGAGATTGACACGTATCTTGTAGTTTTTTA
shMucin2_4	GATCCCCGCGTCCATAACAACGACCTTCAAGAGAAGGTCGTTGTTATGGACGCTTTTTA

**Table 2:** Primers used for qRT-PCR.

Gene Name	Forward	Reverse
*Mucin2*	GGTGAGGAGGTGTACAACGG	CAGCCACCAAGTCTCGTTCT
*L1*	TCGCCCTATGTCCACTACACCT	ATCCACAGGGTTCTTCTCTGGG
*GAPDH*	GTCTCCTCTGACTTCAACAGCG	ACCACCCTGTTGCTGTAGCCAA

## Data Availability

The data presented in this study are available on request from the corresponding author.

## Supplementary Materials

The following supporting information can be downloaded at: https://www.mdpi.com/article/10.3390/ijms241713418/s1.
