# Supplementary material for: An Increase in Mucin2 Expression Is Required for Colon Cancer Progression Mediated by L1
Source: Int J Mol Sci. 2023 Aug 30;24(17):13418. doi: 10.3390/ijms241713418 (PMC10488000; doi:10.3390/ijms241713418)
Supplement: Supplementary file 1 [file ijms-24-13418-s001.zip › ijms-2542724-supplementary.pdf]

<u>**Supplementary Material**</u>

An Increase in Mucin2 Expression is Required for Colon Cancer Progression Mediated by L1

Arka Saha [1], Nancy Gavert [1], Thomas Brabletz [2] and Avri Ben-Ze'ev [1*]

[1] Department of Molecular Cell Biology, Weizmann Institute of Science, Rehovot 7610001, Israel; arka.saha@weizmann.ac.il (A.S.); nancy.gavert@weizmann.ac.il (N.G.)

[2] Department of Experimental Medicine I, Nikolaus-Feibiger-Center for Molecular Medicine, University of Erlangen-Nuernberg, 91054 Erlangen, Germany; thomas.brabletz@fau.de

* Correspondence: avri.ben-zeev@weizmann.ac.il;

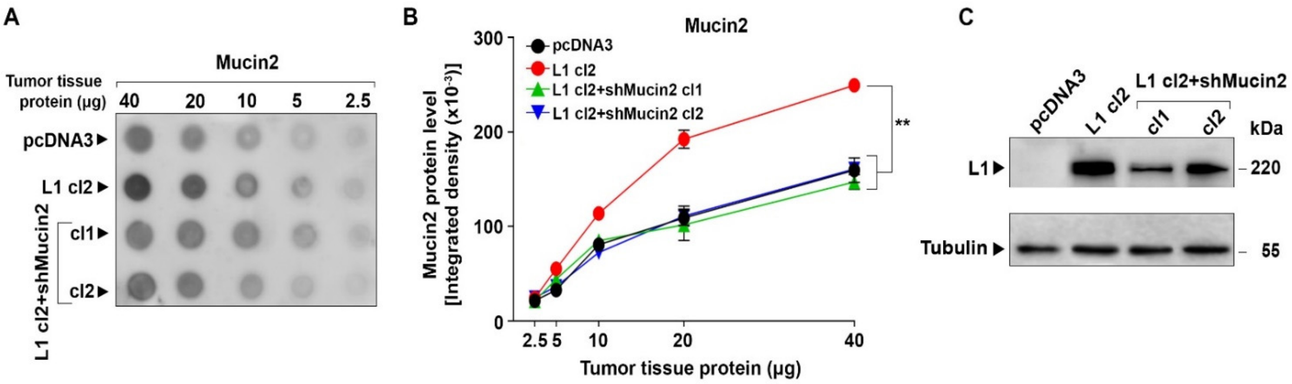

Figure S1: Mucin2 and L1 protein levels in mouse tumor tissue generated by injecting mice with pcDNA3, L1 cl2, and L1 cl2+shMucin2 cl1 and cl2 cells. (**A**): Proteins were extracted from the tumor tissues generated by injecting colon cancer cells expressing pcDNA3, L1, L1+shMucin2 cl1 and cl2 and analyzed for Mucin2 protein level by quantitative dot blot analysis using Mucin2 antibodies. (**B**): Densitometric analysis of the data shown in (A) for Mucin2. (**C**): Analysis of L1 levels by western blotting. Tubulin served as loading control. (** $p < 0.01$). Note: Mucin2 levels remained low in the tumor tissue generated by L1+shMucin2 cl1 and cl2 three weeks after injecting the cells.
